# Advanced Nanoenabled Microalgae Systems: Integrating Oxidative Stress-Induced Metabolic Reprogramming and Enhanced Lipid Biosynthesis for Next-Generation Biofuel Production

**DOI:** 10.1021/acsabm.5c00300

**Published:** 2025-04-09

**Authors:** Luis Pablo Salmeron Covarrubias, Kavitha Beluri, Yasaman Mohammadi, Nusrat Easmin, Oskar A. Palacios, Hamidreza Sharifan

**Affiliations:** † Department of Earth, Environmental and Resource Sciences, University of Texas at El Paso, El Paso, Texas 79968, United States; ‡ Environmental Science and Engineering Program, University of Texas at El Paso, El Paso, Texas 79968, United States; § Faculty of Chemical Sciences, Universidad Autonoma de Chihuahua, Circuito Universitario S/N, Campus UACH II, Chihuahua, Chih 31125, Mexico; ∥ Department of Chemistry and Biochemistry, University of Texas at El Paso, 500 W University Ave, El Paso, Texas 79968, United States

**Keywords:** ZnO NPs, lipid biosynthesis, biomass productivity, catalase activity, biofuel

## Abstract

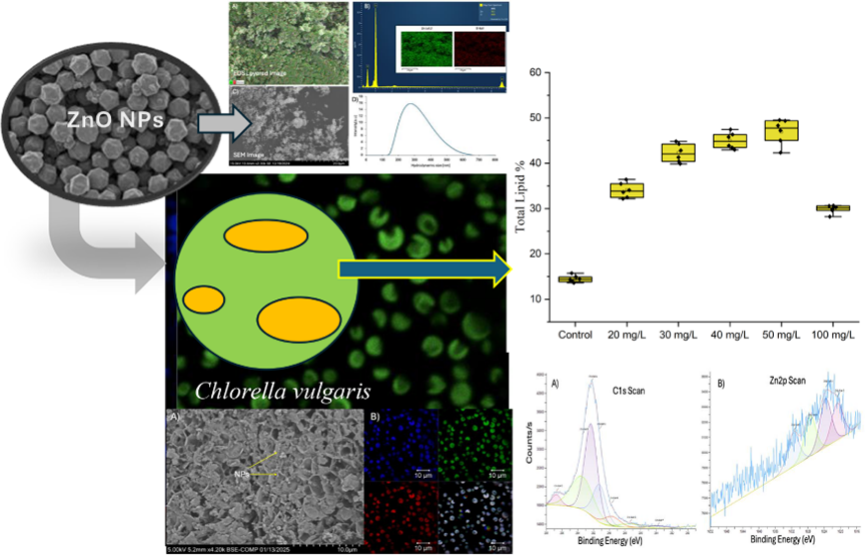

The growing demand for renewable energy has positioned microalgae, such as *Chlorella vulgaris*, as a promising feedstock for sustainable biofuel production. Leveraging nanotechnology, this study explores the multifaceted impacts of zinc oxide (ZnO) nanoparticles (NPs) on *C. vulgaris*, focusing on lipid biosynthesis, oxidative stress, biomass productivity, and photosynthetic pigment retention. The morphology of NPs and algae and their interactions were extensively studied using scanning electron microscopy (SEM), confocal microscopy, energy-dispersive X-ray spectroscopy (EDS), and X-ray photoelectron spectroscopy (XPS). The ZnO NP-enabled microalgae system enhanced lipid accumulation to as high as 48% at 50 mg/L. Biomass production and pigment content remained stable within the applied dose of NPs (20–50 mg/L), highlighting the resilience of *C. vulgaris* under NP exposure. However, at 100 mg/L, photosynthetic efficiency was disrupted, pigment content was reduced, and lipid yield declined to 30%. The enzymatic activity of catalase (CAT) revealed significant upregulation at higher ZnO NP concentrations, further corroborating the stress-induced metabolic shifts. This study also introduced a model for the Biofuel Suitability Score (BSS), which integrates lipid content, biomass productivity, oxidative stress levels, and pigment retention to identify the optimal conditions for biofuel production. The BSS peaked at moderate ZnO NP concentrations (30–50 mg/L), indicating a balance between lipid biosynthesis and cellular integrity. Beyond this threshold, oxidative damage compromises the biofuel potential, emphasizing the critical need for precise control of NP exposure. These findings highlight the potential of ZnO NPs to induce lipid accumulation through targeted stress modulation while maintaining biomass quality, advancing the application of nanotechnology in sustainable bioenergy systems. This study provides a scalable framework for integrating nanotechnology into renewable energy.

## Introduction

The growing global demand for renewable energy has driven significant interest in microalgae as a sustainable feedstock for biofuel production.^1^ Genera of green microalgae like *Chlorella*, *Scenedesmus*, *Nannochloropsis*, and *Chlorococcum* have been mainly used for biofuel production.^2^ Nonetheless, *Chlorella* is recognized as one of the main microalgae genera for biofuel production since it can accumulate more than 55% of lipids.^3^ Additionally, microalgae such as *C. vulgaris* offer several advantages over conventional crops,^4^ including higher lipid productivity,^5^ rapid growth rates, and the ability to thrive in nonarable land using minimal freshwater resources.^6^ However, optimizing lipid accumulation in microalgae remains a critical challenge,^7,8^ as baseline conditions typically prioritize biomass growth over lipid biosynthesis. Recent studies have shown that controlled environmental stress can effectively redirect metabolic pathways in microalgae to enhance lipid accumulation,^9,10^ making this approach a promising strategy for improving biofuel yields.

Nanotechnology has emerged as a tool for inducing targeted stress responses in microalgae,^9^ with zinc oxide (ZnO) nanoparticles (NPs) gaining attention due to their unique physicochemical properties and biological interactions.^11,12^ ZnO NPs are known to generate reactive oxygen species (ROS) in aqueous environments,^13^ which can induce oxidative stress in microalgae,^14^ triggering lipid biosynthesis as part of the cellular stress response. For example, in 50 ppm ZnO NPs in *Chlorella* sp., cultures positively affected neutral lipids and triacylglycerol production without a complete inhibition growth.^15^ However, the effects of ZnO NPs are concentration- and microalga species-dependent, and excessive stress can impair photosynthetic efficiency, reduce pigment content, and compromise biomass productivity, ultimately diminishing biofuel potential. For example, in *C. vulgaris* cultures, concentrations of ZnO NPs higher than 2.5 ppm decrease cell growth and photosynthetic pigment content, producing a deformation of cellular morphology.^16^ Nonetheless, Kumar et al. (2014) reported that concentrations around 5 ppm of ZnO NPs increased chlorophyll content in *Chlorella* sp.^13^ Despite these challenges, there is a limited understanding of the optimal ZnO NP concentrations required to balance lipid accumulation with overall cellular health in *C. vulgaris*.

This study investigates the multifaceted effects of ZnO nanoparticles on *C. vulgaris* by evaluating key parameters such as lipid content, biomass production, pigment concentration, and oxidative stress response. By integrating these variables into a comprehensive biofuel suitability score, this research aims to identify the optimal ZnO NP concentration for maximizing biofuel production. The findings provide critical insights into the interplay between nanoparticle-induced stress and microalgal metabolism, offering a scalable and efficient approach for enhancing lipid yields while maintaining the physiological integrity of microalgae. This work advances the application of nanotechnology in sustainable energy production and lays the foundation for further exploration of nanoparticle–algal interactions in biofuel development. The Biofuel Suitability Score (BSS) is a novel approach proposed in this study, serving as a method to assess the biofuel production potential of *C. vulgaris* under varying concentrations of ZnO nanoparticles. This score integrates crucial biochemical parameters, such as lipid accumulation, biomass productivity, and photosynthetic pigment content, to provide a comprehensive evaluation of biofuel suitability. The necessity for designing this score arises from balancing oxidative stress management with optimal lipid production as traditional assessments often overlook such multifaceted interactions. By quantifying these diverse parameters into a single metric, BSS enhances our understanding of how ZnO nanoparticles influence biomass quality and biofuel yield, ultimately guiding more effective strategies for sustainable biofuel production from microalgae. While previous studies have explored the impact of ZnO NPs on microalgal lipid accumulation, biomass productivity, and oxidative stress responses,^15,17^ they have primarily focused on broad stress-induced lipid biosynthesis mechanisms without integrating a comprehensive assessment framework.

The BSS model incorporates multiple biochemical and physiological parameters, such as lipid accumulation, biomass production, oxidative stress, and photosynthetic efficiency, to optimize the biofuel production conditions. Unlike prior works that have examined individual stress responses in microalgae, our approach systematically evaluates the trade-offs between stress-induced lipid enhancement and overall cell viability. Furthermore, the research uniquely quantifies the interactions between ZnO NPs and *C. vulgaris* using X-ray photoelectron spectroscopy (XPS), scanning electron microscopy (SEM), and confocal microscopy, providing deeper mechanistic insights into nanoparticle–algal interactions. These distinctions position our study as a framework for integrating nanotechnology into biofuel production while ensuring biomass sustainability.

## Materials and Methods

### Materials and Reagents

Zinc oxide (ZnO) nanoparticles (99%) were obtained from US Research Nanomaterials. Xylenol orange, HPLC-grade methanol, sodium carbonate anhydrate, Folin–Ciocalteu phenol reagent, gallic acid hydrate, ethanol, pyrogallol, guaiacol, sulfuric acid, and trichloroacetic acid (TCA) solution were procured from Thermo Fisher Scientific (USA). Anthrone ACS solution, nicotinamide adenine dinucleotide (NADH), and thiobarbituric acid (TBA) were purchased from Sigma-Aldrich (USA). All chemicals were of analytical grade (≥99% purity) and used as received without further purification. *C. vulgaris* (UTEX 2714) and the Blue-Green medium (BG-11) were procured from the Culture Collection of Algae at the University of Texas at Austin (UTEX), USA. The BG-11 medium was prepared with the following chemical composition (mg/L): NaNO_3_ (1500), K_2_HPO_4_ (40), MgSO_4_·7H_2_O (75), CaCl_2_·2H_2_O (36), Na_2_CO_3_ (20), EDTA-Na_2_ (1), ferric ammonium citrate (6), citric acid (6), H_3_BO_3_ (2.86), MnCl_2_·4H_2_O (1.81), ZnSO_4_·7H_2_O (0.222), NaMoO_4_·2H_2_O (0.39), CuSO_4_·5H_2_O (0.079), and Co(NO_3_)_2_·6H_2_O (0.0494).

### Material Characterizations

#### Energy-Dispersive X-Ray Spectroscopy (EDS)

Energy-dispersive X-ray spectroscopy (EDS) was conducted to analyze the elemental composition and spatial distribution of ZnO NPs. Samples were examined using a HITACHI SU3500 scanning electron microscope (SEM) under high vacuum conditions (10^–6^ Torr). To ensure high spatial resolution and minimize sample damage, an accelerating voltage of 15 kV and a beam current of 1 nA were employed, with a working distance of 10 mm for optimal X-ray collection.^20^ Regions of interest were identified, and elemental spectra were collected by using the Oxford Instruments X-Max EDS detector. Major elements, including Zn and O, were analyzed. Elemental mapping was performed at a resolution of 1024 × 1024 pixels on a 250 μm scale, providing detailed spatial differentiation of the elements. The dwell time was set to 100 ms per pixel to optimize the balance between the signal-to-noise ratio and data acquisition time.

#### Characterization via High-Resolution Transmission Electron Microscopy

In this study, the morphological characteristics of the nanoparticles were analyzed using a JEM-3200FS field emission transmission electron microscope (TEM) operating at an accelerating voltage of 300 kV. Ten mg/L of ZnO NPs were dispersed in an ultrasonic bath for 10 min and then deposited as three sequential drops onto a carbon-coated copper grid, allowing each drop to dry under ambient conditions. Once fully dried, the grid was placed in the microscope’s vacuum chamber for high-resolution imaging.

#### X-Ray Photoelectron Spectroscopy (XPS) Analysis

Algal subsamples exposed to ZnO NPs were collected from treatments of 50 mg/L via centrifugation at 5000 rpm for 10 min, followed by three washes with deionized water (DI) to remove any unbound NPs. The resulting biomass was freeze-dried and finely ground to obtain a homogeneous powder. A thin layer of the sample was pressed onto an indium foil substrate to ensure optimal conductivity and minimal charging effects during XPS measurements. The prepared samples were then transferred to the XPS vacuum chamber and analyzed under ultrahigh vacuum conditions to investigate the surface interactions between *C. vulgaris* and ZnO NPs. X-ray photoelectron spectroscopy (XPS) was performed using a Kratos Axis Ultra DLD spectrometer equipped with a monochromated Al Kα X-ray source operating at 150 W. High-resolution spectra of the binding energy (BE) were acquired for the C 1s and Zn 2p regions to examine the interaction between *C. vulgaris* and ZnO NPs.

#### Confocal Microscopy Analysis

A Laser Scanning Microscope (LSM) 700 (Zeiss, New York, NY) was utilized for the microscopic analysis.^18^ Even though the algae samples were nonstained, high-resolution digital fluorescent confocal microimages were captured using three fluorescent channels: blue (DAPI), green (Alexa-488), and red (Alexa-568). Moreover, an EC Plan-Neofluar 20x objective lens was used with a pinhole adjusted to 1 Airy Unit (AU) per channel, a laser power of 5, and a consistent Gain Master.^19^ To capture images of the ZnO nanoparticles, a phase-contrast approach was included to obtain additional bright-field images and the three fluorescent channels with the same settings as those mentioned above. For the acquisition and analysis of confocal microimages, ZEN 2009 software was utilized (Zeiss).

#### Dynamic Light Scattering (DLS)

The particle size distribution and zeta potential of 20 mg/L ZnO NPs were analyzed using a Malvern Nano ZS Instrument (Malvern Instruments GmbH, Germany). ZnO NPs were dispersed in deionized water and subjected to ultrasonication using a probe sonicator (Model: QSonica Q500) at 50% amplitude for 15 min.^20^ This ensured the uniform dispersion of nanoparticles and minimized agglomeration prior to measurement.

#### Experimental Design

*C. vulgaris* cultures (10^6^ cells/mL) were grown in triplicate under controlled laboratory conditions with ZnO NP concentrations of 0 (control), 20, 30, 40, 50, and 100 mg/L. Each treatment group contained 250 mL of algal culture maintained in sterile 500 mL Erlenmeyer flasks. ZnO NPs were prepared as stock solutions, sonicated for 30 min to ensure homogeneity, and added to the respective treatment flasks at the beginning of the experiment.

#### Culture Conditions

The cultures were maintained under standardized conditions to ensure consistent growth across all treatment’s cultures that were grown in BG-11 prepared using sterile distilled water, where the temperature was set at 25 ± 1 °C and the light intensity was 243 μmol photons m^–2^ s^–1^ provided by full-spectrum lamps under a rotation of a 16:8 h light–dark cycle.^5^ The reactors were subjected to continuous agitation at 150 rpm on an orbital shaker to ensure a uniform distribution of nanoparticles and to prevent sedimentation of the culture. I suggest adding here the duration of the experiment.

#### Biomass Production

Biomass production was quantified as dry cell weight (DCW) per liter at the end of a 7-day cultivation period. A 50 mL aliquot of each culture was collected and centrifuged at 5000 rpm for 10 min at 4 °C to pellet the biomass. The resulting pellet was washed twice with deionized water to remove residual medium and ZnO NPs, ensuring accurate measurement of the biomass. The washed biomass was then transferred to preweighed aluminum trays and dried at 60 °C for 48 h until a constant weight was achieved. Biomass production was calculated using eq 1:
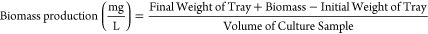
1

#### Chlorophyll and Carotenoid Content

The quantification of photosynthetic pigments, including chlorophyll a, chlorophyll b, and carotenoids, was conducted under the specified growth conditions following standard methodologies. A 10 mL sample of *C. vulgaris* culture, harvested during the exponential growth phase (day 11), which occurred during the cultivation period, was centrifuged at 7000 rpm for 10 min to isolate the cell biomass. The supernatant was carefully removed, and the cell pellet was rinsed with distilled water to eliminate any residual medium. Subsequently, 10 mL of methanol was added to the rinsed biomass, and the mixture was vigorously agitated to enhance the pigment extraction. The samples were then incubated in a water bath at 60 °C for 15 min to facilitate the efficient release of pigments from the cells. Following incubation, the mixture was centrifuged at 4000 rpm for 5 min, and the resulting supernatant, containing the extracted pigments, was collected for optical density (OD) analysis. Chlorophyll a and chlorophyll b concentrations were determined by measuring the OD at 665 and 652 nm, respectively. To assess carotenoid content, the remaining biomass was resuspended in 10 mL of distilled water, stored at 4 °C for 1 h, and subsequently centrifuged again at 4000 rpm for 5 min. The supernatant was collected, and its OD was measured at 470 nm. Methanol was used as the blank control for all of the spectrophotometric measurements to ensure precision.

The concentrations of chlorophyll a (Chl a), chlorophyll b (Chl b), and carotenoids were calculated using eqs 2–4 tailored for each pigment’s absorbance characteristics.
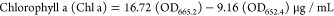
2
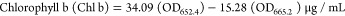
3
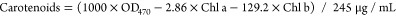
4

#### Lipid Content Analysis

The lipid content of *C. vulgaris* was determined using the adapted Folch method.^21^ A chloroform/methanol mixture (2:1, v/v) was added to the algal biomass at a solvent-to-biomass ratio of 20:1, followed by thorough homogenization to ensure complete lipid extraction. To facilitate phase separation, 0.2 volumes of distilled water or 0.9% NaCl solution were added to the homogenate, which was then centrifuged at 3000 rpm for 10 min. The upper aqueous phase was carefully removed, and the lipid-rich lower phase was transferred to preweighed vials.

The extracted lipid phase was further purified by washing with a 0.9% NaCl solution to remove impurities. The chloroform in the lipid extract was subsequently evaporated under a stream of nitrogen gas to prevent lipid oxidation. The remaining dried lipid extracts were weighed, and the lipid content was expressed as a percentage of the initial dry weight of *C. vulgaris*. All extractions were performed in triplicate to ensure reproducibility and accuracy of the results.

#### Catalase (CAT) Activity Assay

Catalase (CAT) activity was measured to assess the oxidative stress levels in *C. vulgaris* under varying concentrations of ZnO NPs. The enzymatic activity was determined spectrophotometrically by monitoring the decomposition of hydrogen peroxide (H_2_O_2_) at 240 nm. A 50 mL aliquot of each algal culture was centrifuged at 5000 rpm for 10 min at 4 °C to obtain a cell pellet. The cell pellet was washed twice with phosphate buffer (50 mM, pH 7.0) to remove the residual medium and ZnO NPs. The washed pellet was resuspended in 5 mL of ice-cold phosphate buffer and homogenized using a tissue grinder to rupture the cells and release intracellular enzymes. The homogenate was centrifuged at 12 000 rpm for 15 min at 4 °C, and the supernatant was collected as the crude enzyme extract.

The CAT activity assay was performed in triplicate for each sample. The reaction mixture consisted of 2 mL of freshly prepared 20 mM hydrogen peroxide (H_2_O_2_) in phosphate buffer (pH 7.0) and 0.1 mL of the crude enzyme extract. The enzymatic reaction was initiated by adding the enzyme extract to the reaction mixture in a quartz cuvette. The decrease in absorbance at 240 nm, corresponding to the decomposition of H_2_O_2_, was monitored spectrophotometrically at 10 s intervals for 1 min using a UV–vis spectrophotometer. The enzymatic activity was expressed as micromoles of H_2_O_2_ decomposed per minute per milligram of protein using eq 5:
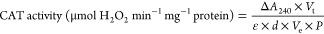
5

where:

Δ*A*_240_: Change in absorbance at 240 nm per minute

*V*_t_: Total reaction volume (2.1 mL)

ε: Molar extinction coefficient for H_2_O_2_ at 240 nm (43.6 M^–1^ cm^–1^)

*d*: Path length of the cuvette (1 cm)

*V*_e_: Volume of enzyme extract used (0.1 mL)

*P*: Protein concentration in the enzyme extract (mg/mL), determined using the Bradford assay

#### Biofuel Suitability Score (BSS) Modeling

To evaluate the biofuel production potential of *C. vulgaris* under different ZnO NP concentrations, a Biofuel Suitability Score (BSS) was developed. The methodology for calculating the BSS is outlined below. The following parameters were considered in the BSS calculation: the lipid percentage (*L*), which indicates the fraction of the total biomass composed of lipids and is a critical determinant of biofuel yield; the biomass productivity (*G*), which represents the overall growth of *C. vulgaris* and is essential for scaling biofuel production; and the oxidative stress (*S*), assessed via catalase (CAT) activity, which is a proxy for ROS levels. Excessive stress impairs cellular health and reduces biofuel quality and the photosynthetic pigment (*P*), which includes chlorophyll a, chlorophyll b, and carotenoid content, reflecting photosynthetic efficiency and overall cellular functionality.

To combine the parameters into a single score, all values were normalized to a scale of 0–1. The normalized values were calculated as follows:

, where *L*_max_ is the highest lipid percentage observed.

, where *G*_max_ is the maximum biomass observed.

, where *S*_max_ is the highest catalase activity observed. Stress is penalized in the final score by using 1–*S*_norm_.

, where *P*_max_ is the highest pigment concentration (chlorophyll a, b, or carotenoids) observed.

The BSS was calculated (eq 6) using the weighted sum of normalized parameters, with stress contributing as a penalizing factor. *L*_norm_, *G*_norm_, and *P*_norm_ indicate the normalized lipid percentage, biomass, and pigment content, respectively. *S*_norm_ represents the normalized stress (CAT activity). Table 1 shows the weights assigned to each parameter based on its relative importance in determining biofuel suitability.
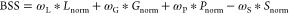
6

**Table 1:** Parameters Applied in Biofuel Suitability Score (BSS) Modeling

Parameter	Weight (w)	Rationale
Lipid Percentage (**ω**_L_)	0.4	The primary feedstock for biodiesel.
Biomass Productivity (**ω**_G_)	0.3	Raw material for biofuel production.
Photosynthetic Pigments (**ω**_P_)	0.2	Photosynthetic efficiency and cellular health.
Oxidative Stress (**ω**_S_)	0.1	Excessive stress can reduce the quality and efficiency of biofuels.

The BSS method integrates multiple biochemical and physiological parameters to provide a holistic evaluation of biofuel potential in *C. vulgaris*. BSS quantifies the balance among lipid biosynthesis, oxidative stress, and cellular viability. The accuracy of the BSS approach was validated by correlating the lipid yield and biomass productivity with oxidative stress indicators (CAT activity), ensuring that the score reliably reflects the optimal nanoparticle concentration for biofuel production.

#### Statistical Analysis

All measurements were performed in triplicate using SPSS (19.0), and the data were expressed as mean ± standard deviation.^22^ Significant differences among treatments were determined using one-way ANOVA followed by Tukey’s post hoc test (*p* < 0.05).

## Results and Discussion

### Elemental Composition and Morphology Analysis of ZnO Nanoparticles and Microalgae

**Figure 1:**
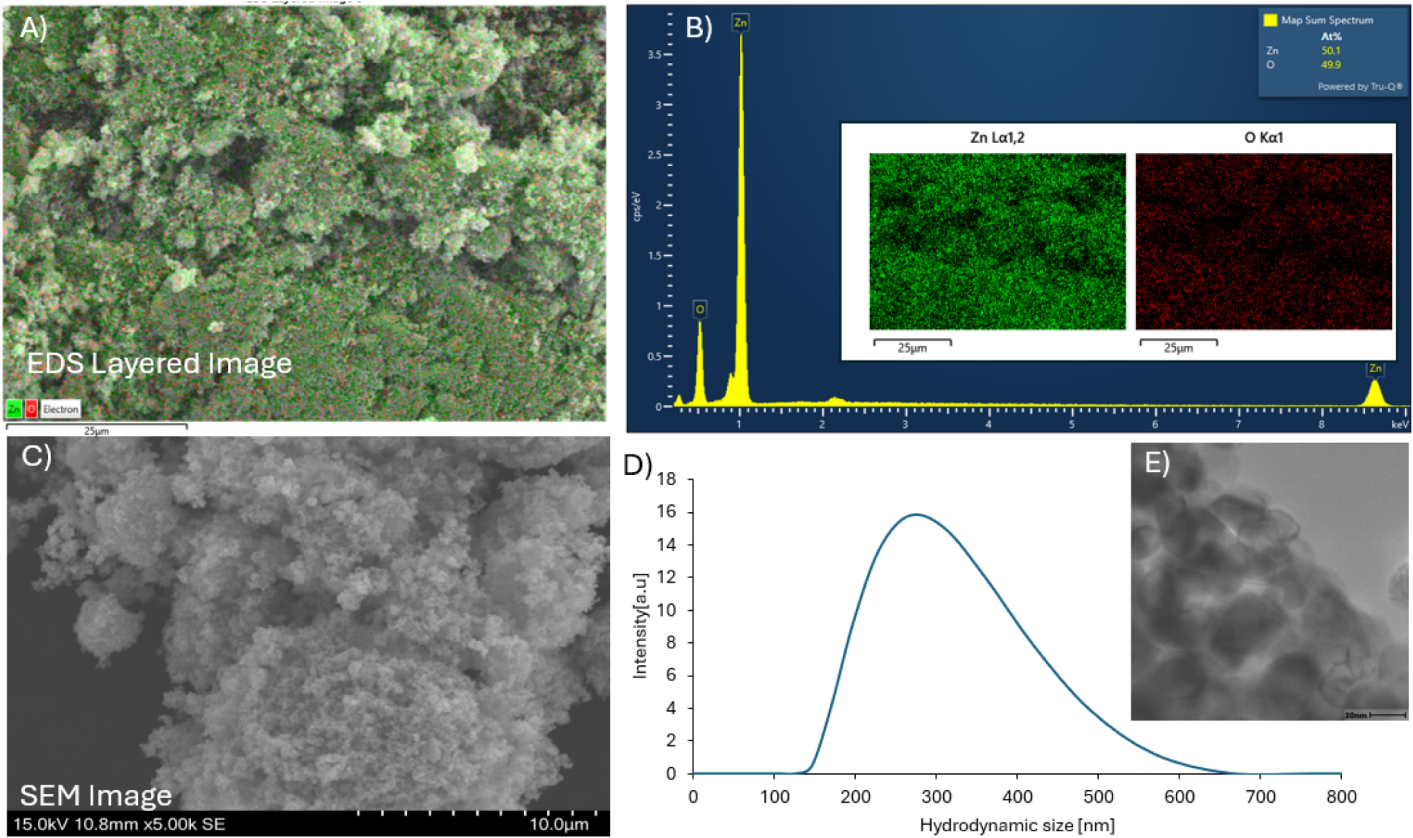
Characterization of ZnO nanoparticles: (A) EDS layered image showing the spatial distribution of Zn and O elements; (B) EDS spectrum confirming the elemental composition with inset maps of Zn and O; (C) SEM image highlighting the surface morphology of ZnO NPs; (D) hydrodynamic size distribution of ZnO NPs obtained via dynamic light scattering (DLS); and (E) TEM image of the ZnO NPs.

The elemental distribution of the ZnO NPs was confirmed through energy-dispersive spectroscopy (EDS). Figure 1, Panel A, presents the EDS layered image, demonstrating a uniform distribution of zinc (Zn) and oxygen (O) throughout the sample. The elemental mapping (inset of Panel B) further corroborates the consistent presence of Zn and O, with the green and red signals corresponding to Zn and O, respectively. The EDS spectrum (Panel B) indicates strong peaks for Zn and O, confirming their dominance in the sample, with an atomic percentage of 50.1% for Zn and 49.9% for O. These results confirm the high purity of the ZnO NPs, consistent with the expected stoichiometry for ZnO. The SEM image in Panel C reveals that ZnO NPs exhibit an irregular and slightly aggregated morphology. The nanoparticles appear to form clusters with varying shapes and sizes, characteristic of ZnO synthesized at the nanoscale. The SEM analysis also indicates a rough surface topology, which could enhance their surface reactivity and adsorption capacity. Further, Panel E confirms the irregular shape and the size range between 20 and 30 nm. This morphology aligns with the characteristics of ZnO in environmental applications, where high surface area and irregularity promote interaction with contaminants. DLS analysis (Panel D) shows the hydrodynamic size distribution of ZnO NPs. The size distribution indicates a primary peak centered around 300 nm, suggesting the presence of nanoaggregates. The zeta potential of the ZnO NPs was measured at 16.42 mV. These results are critical for understanding the behavior of ZnO NPs in aqueous algae systems, where aggregation can influence their dispersion, reactivity, and interactions with algae cells.

The characterization results highlight the key physical and chemical properties of ZnO NPs, demonstrating their potential for environmental applications. The uniform elemental distribution and high purity, confirmed through EDS analysis, establish ZnO as a suitable material for nanoremediation. The irregular morphology and nanoaggregates observed in the SEM and DLS analyses suggest high surface area and potential for enhanced reactivity, critical contaminant adsorption, and catalytic processes. However, the aggregation observed in the DLS analysis underscores the need for further optimization to improve nanoparticle dispersion, as aggregation could reduce surface accessibility and efficacy. These findings provide a foundation for understanding the role of ZnO NPs in complex environmental systems, particularly in interactions with contaminants such as PFOA, and pave the way for optimizing their application in water treatment and remediation strategies.

**Figure 2:**
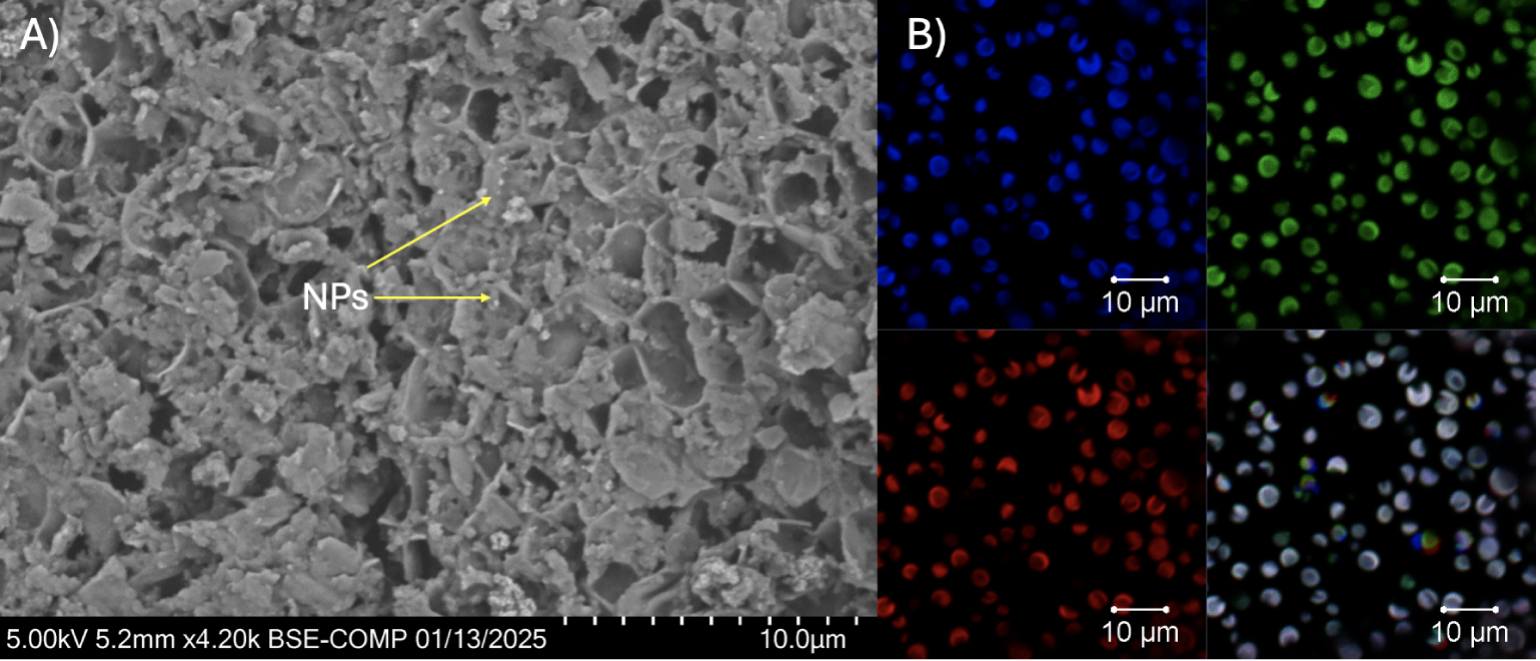
Microscopic characterization of *C. vulgaris* culture. (A) Scanning electron microscopy (SEM) of 100 mg/L ZnO NPs exposed to cell culture, revealing the surface morphology of cells. (B) Confocal laser scanning microscopy (CLSM) of 20 mg/L ZnO NPs exposed to cell culture: autofluorescence of chlorophyll (blue channel) highlights the distribution of photosynthetic pigments, and green fluorescence confirms the structural integrity and uniform spatial distribution of the algal cells.

Figure 2A shows the SEM image of exposed *C. vulgaris* culture to 100 mg/L NPs. This concentration was selected for SEM analysis, as the lower concentration was challenging to track the presence of NPs. The porous surface observed in *C. vulgaris* in Figure 2A does not indicate complete cell rupture but is rather a result of nanoparticle interaction and stress-induced morphological changes. Exposure to ZnO NPs can lead to alterations in the cell wall structure due to oxidative stress, causing surface roughness and increased porosity. Several studies have reported similar effects when microalgae are exposed to metal oxide nanoparticles, where the stress response leads to modifications in the extracellular polysaccharide layer and cell wall integrity. The absence of debris or contamination in the culture medium underscores the sample’s purity. The scale bar (10 μm) in Figure 2A verifies the typical cell diameters of 3–8 μm, consistent with known dimensions of *C. vulgaris* with NPs that are distributed evenly on the surface. These findings validate the health and suitability of the culture for further experimental investigations, particularly in exploring interactions with ZnO NPs.

Confocal laser scanning microscopy, as shown in Figure 2, was employed to characterize the *C. vulgaris* culture upon exposure to 20 mg/L ZnO NPs, revealing uniform, spherical cells with dense and even distribution, indicative of robust growth. The blue channel (Figure 2B) highlights the autofluorescence of chlorophyll, reflecting the presence of active photosynthetic pigments critical for cellular metabolism. The green channel (Figure 2B) confirmed the structural integrity of the cells, showcasing a consistent and uniform fluorescence pattern. This pattern supports the observation of healthy, metabolically active cells with no evidence of clustering, aggregation, or biofilm formation.

### Biomass Analysis

**Figure 3:**
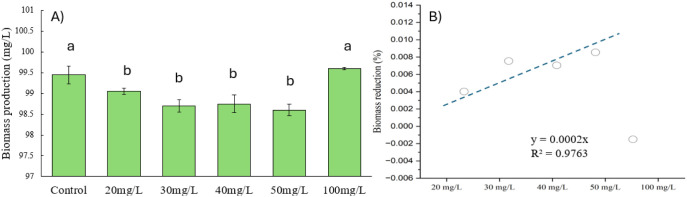
(A) Biomass production of *C. vulgaris* exposed to varying concentrations of ZnO nanoparticles (0, 20, 30, 40, 50, and 100 mg/L). (B) Calculated biomass reduction as a function of ZnO NP concentration. Error bars represent standard deviations, and different letters indicate statistically significant differences (*p* < 0.05).

The biomass production of *C. vulgaris* under varying ZnO NP concentrations (control, 20–100 mg/L) is presented in Figure 3A. The data reveal clear trends in biomass production, with notable reductions at moderate ZnO NP concentrations and a surprising increase at higher concentrations. The control group, which was not exposed to ZnO NPs, exhibited the highest biomass production (99.5 mg/L), indicating optimal growth under standard conditions. Biomass production showed a slight but consistent decline at 20–50 mg/L ZnO concentrations, ranging from 98.6 to 98.75 mg/L. This reduction suggests mild stress imposed by the ZnO NPs, likely due to ROS generation that disrupts cellular metabolism. However, the decline was not severe, highlighting the inherent resilience of *C. vulgaris* to nanoparticle-induced stress. The tolerance of microalgae to different ZnO NP concentrations has been reported to be related to the cell wall structure of the specific microalgae. For example, in the microalgae *Dunaliella salina*, the absence of a cell wall produces high sensitivity to low concentrations of ZnO since these NPs can easily enter the cell and influence growth.^23^ On the other hand, *C. vulgaris* presents a rough cell wall that can induce a physical interaction with ZnO NPs, avoiding the entry of ZnO NPs or Zn ions into the cell.^24^ Nonetheless, under high concentrations of ZnO, the surface area of microalga cells is fully occupied by NPs, thus reducing the interface area for nutrient exchange, as was observed in *C. vulgaris* in the presence of nickel oxide nanoparticles.^25^

Interestingly, at 100 mg/L ZnO, biomass production significantly increased (*p* < 0.05) to 100 mg/L, surpassing the control. This unexpected trend may be attributed to the aggregation of these NPs, which occurs and decreases the release rate of Zn ions to the medium, thus reducing the negative effect on the microalgae cells^26^ being the effect of ZnO NPs only observed on pigment production and a hormetic effect, where high stress levels stimulate protective adaptive responses, such as the upregulation of antioxidant enzymes, enabling *C. vulgaris* to mitigate oxidative damage. Despite this increase in biomass, earlier data on chlorophyll and carotenoid contents at 100 mg/L ZnO indicate compromised photosynthetic efficiency and reduced cellular quality, suggesting that this growth may not translate into optimal biofuel feedstock.

The slight biomass reductions at 20–50 mg/L ZnO are consistent with the generation of ROS that hinders growth while still allowing *C. vulgaris* to maintain cellular functions. This concentration range appears to balance the trade-offs between the biomass yield and lipid accumulation, making it favorable for biofuel applications. Conversely, while biomass at 100 mg/L is high, the stress-related decline in the pigment and lipid content significantly limits its suitability for biofuel production.

Figure 3B highlights the relationship between the ZnO NP concentration and calculated biomass reduction, showing a linear trend up to 50 mg/L. This supports the conclusion that moderate ZnO concentrations impose manageable stress on *C. vulgaris*, enabling the cells to maintain a balance between growth and stress responses. These findings suggest that the optimal ZnO concentration range for biofuel production lies between 20 and 50 mg/L, where lipid accumulation is enhanced without severely compromising biomass yield or quality. Further research should focus on elucidating the molecular mechanisms driving these responses and optimizing conditions for scalable biofuel production.

### Chlorophyll and Carotenoid Contents

**Figure 4:**
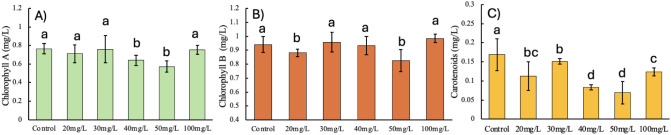
Effects of ZnO nanoparticle concentrations (0, 20, 30, 40, 50, and 100 mg/L) on photosynthetic pigments in *C. vulgaris*: (A) chlorophyll a content, (B) chlorophyll b content, and (C) carotenoids content. Error bars represent standard deviations, and different letters above bars indicate statistically significant differences (*p* < 0.05) among treatments.

As illustrated in Figure 4, the chlorophyll and carotenoid contents of *C. vulgaris* under different ZnO nanoparticle concentrations reveal significant trends linked to the photosynthetic performance and stress response. Chlorophyll a levels (Figure 4A) remained stable in the control and at 20–30 mg/L ZnO, with values around 1.0 mg/L. However, a significant decline was observed at 40 and 50 mg/L, indicating that moderate ZnO concentrations negatively impact photosynthetic efficiency. A decrease of photosynthetic pigments in the microalga *Chlorosarcinopsis* sp. due to the presence of 50 ppm ZnO NPs was also reported by Vasistha and coworkers,^27^ being explained by the shading effect produced by the high NP concentration, which affects the photosynthetic activity of the microalga. Nonetheless, in our work, a concentration of 100 mg/L ZnO NPs induces a partial recovery in chlorophyll a levels. This could be explained by a possible aggregation of ZnO NPs at high concentrations, which reduces the rate of Zn ion release and allows a better response of microalgae^28^ through stress-induced adaptive mechanisms. Chlorophyll B (Figure 4B) followed a similar trend, with values remaining steady across the control and 20–30 mg/L before declining at 40 and 50 mg/L and recovering slightly at 100 mg/L. The recovery of both pigments at 100 mg/L suggests that while extreme stress disrupts photosynthetic activity, *C. vulgaris* exhibits some capacity for adaptation under severe conditions.

Carotenoid content (Figure 4C), which is crucial for mitigating oxidative stress, showed a more pronounced decline. Starting from 20 mg/L, carotenoid levels dropped progressively, reaching their lowest values at 40 and 50 mg/L. At 100 mg/L, a slight increase was noted, but carotenoid content remained significantly below control levels. A similar effect has been reported in other microalgae like *Spirulina platensis*, in which, independently of the concentration, ZnO NPs tend to reduce chlorophyll, phycocyanin, and carotenoid contents.^29^ This pattern highlights the inability of *C. vulgaris* to maintain adequate carotenoid levels under ZnO-induced stress, particularly at moderate to high nanoparticle concentrations. The decline in carotenoids likely exacerbates oxidative damage, further inhibiting photosynthetic efficiency.

These results underscore the sensitivity of photosynthetic pigments to the ZnO nanoparticle exposure. While 20–30 mg/L ZnO concentrations maintain relatively stable pigment levels, higher concentrations lead to significant reductions, impacting photosynthetic performance and cellular health. This suggests that ZnO concentrations above 30 mg/L may compromise biofuel production by impairing the biomass quality. Maintaining ZnO concentrations within 20–30 mg/L is critical for balancing lipid production, photosynthetic efficiency, and oxidative stress mitigation. Further studies should explore the interplay among pigment content, lipid accumulation, and stress response to optimize biofuel production systems.

### Enzymatic Activities

**Figure 5:**
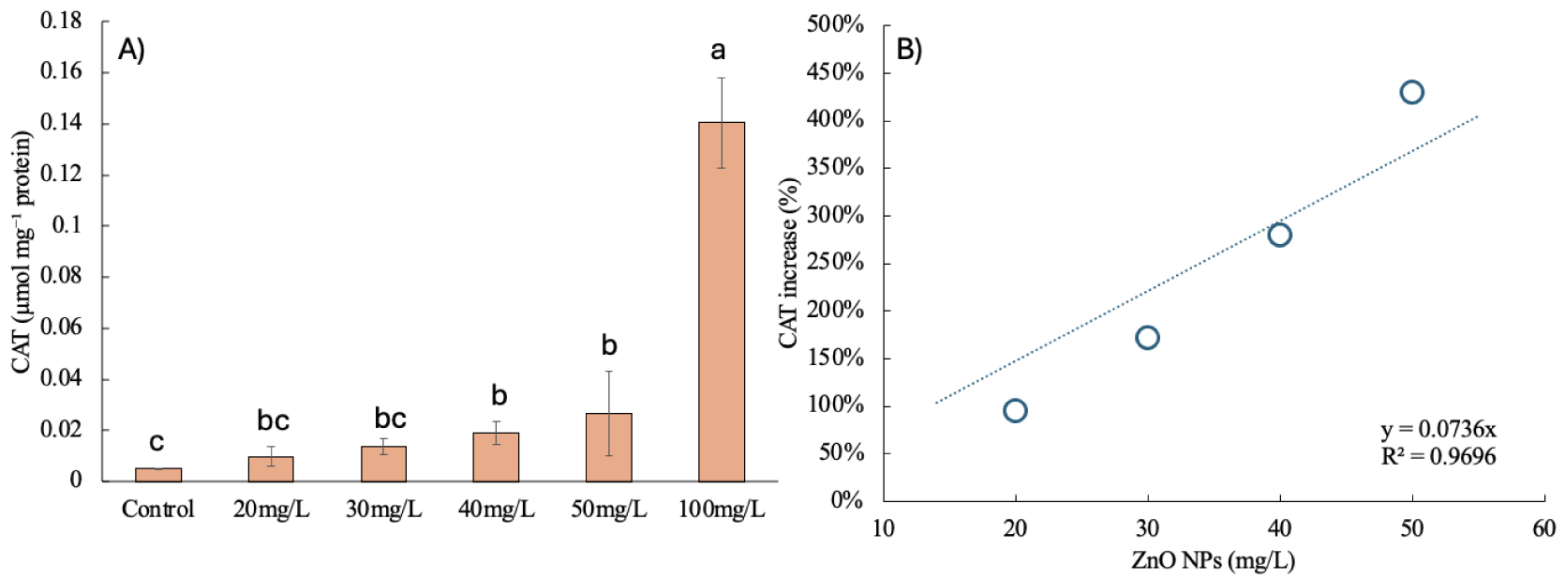
Impact of ZnO nanoparticles on catalase (CAT) activity in *C. vulgaris*: (A) CAT activity (μmol mg^–1^ protein) at varying ZnO NP concentrations (20–100 mg/L) compared to the control, with different letters indicating statistically significant differences (*p* < 0.05). Error bars represent standard deviations. (B) Linear correlation between ZnO NP concentration and percentage increase in CAT activity.

The catalase (CAT) activity of *C. vulgaris* exposed to varying concentrations of ZnO NPs highlights the oxidative stress response triggered by nanoparticle exposure (Figure 5). Under control conditions, CAT activity was minimal (0.005 μmol mg^–1^ protein), reflecting the absence of oxidative stress in the baseline environment. At low to moderate ZnO NP concentrations (20–40 mg/L), CAT activity increased modestly, with values ranging from 0.01 to 0.02 μmol mg^–1^ protein. This suggests that these concentrations induce mild oxidative stress, which the cells can effectively manage through the limited upregulation of antioxidant defenses.

At 50 mg/L ZnO NPs, CAT activity increased further, indicating a more pronounced oxidative stress response. This aligns with reductions in carotenoid content observed at this concentration, as carotenoids play a critical role in scavenging ROS. The sharp spike in CAT activity at 100 mg/L (0.14 μmol mg^–1^ protein) reflects severe oxidative stress, which overwhelms the cell’s antioxidant systems and triggers a strong enzymatic response. While this indicates the activation of stress-adaptive mechanisms, the oxidative burden at this concentration likely disrupts cellular homeostasis, as evidenced by declines in photosynthetic pigments and lipid content. Similar results were reported in the microalga *Haematococcus pluvialis* exposed to different concentrations of ZnO NPs, in which the antioxidant enzyme activity was proportional to the increase of NP concentration, indicating a response to stress by the microalgae cells, but with a negative effect of photosynthetic pigment production.^30^

The linear correlation between the ZnO NP concentration and CAT activity (Figure 5B) reinforces the concentration-dependent nature of oxidative stress induced by the nanoparticles. At low to moderate concentrations (20–40 mg/L), the oxidative stress response remains manageable, allowing cells to sustain photosynthetic efficiency and lipid biosynthesis. However, at higher concentrations (50–100 mg/L), ROS accumulation surpasses the cells’ capacity to maintain cellular integrity, compromising biofuel production potential. In this sense, it has been reported that concentrations of 100 ppm ZnO NPs or more induce a decrease of respiration affecting growth and metabolite production by *Chlorella* sp.^15^

For biofuel applications, maintaining ZnO NP concentrations within the range of 20–40 mg/L minimizes oxidative stress while supporting optimal lipid and pigment production. The addition of low concentrations of metallic nanoparticles to induce lipid production in microalgae is well recognized due to the enhanced effect of NPs on an enzymatic activity like acetyl-coenzyme A carboxylase, which catalyzes the first step in fatty acid biosynthesis.^31^ Beyond this range, oxidative damage reduces biomass quality and lipid yields and induces the peroxidation of preexisting lipids, limiting the practical utility of *C. vulgaris* as a biofuel feedstock. Future studies should investigate strategies to mitigate oxidative stress, such as antioxidant supplementation, to enhance the resilience of *C. vulgaris* under elevated nanoparticle exposure.

### Lipid Content Analysis

**Figure 6:**
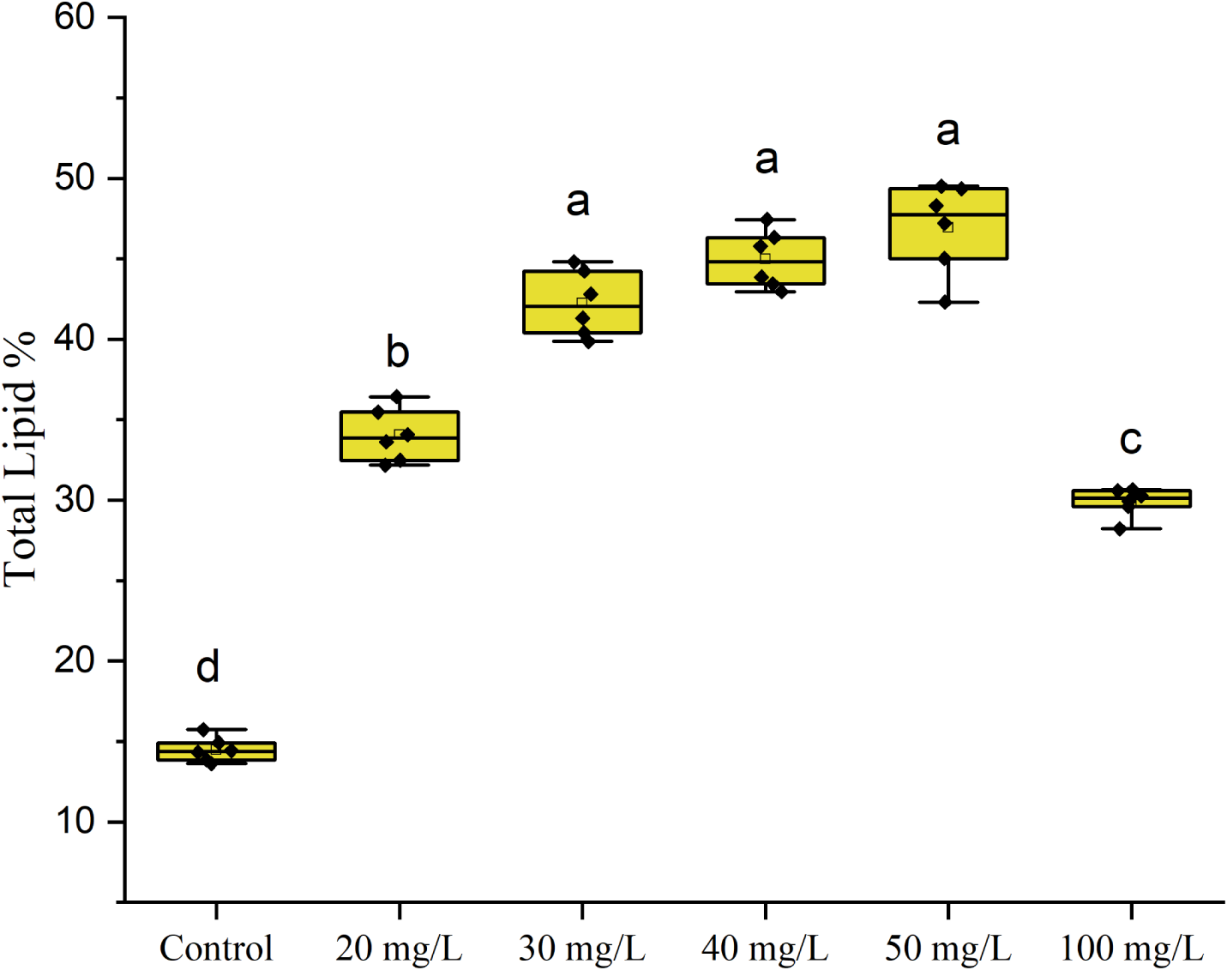
Total lipid content (%) of *C. vulgaris* after exposure to ZnO nanoparticle (0, 20, 30, 40, 50, and 100 mg/L) concentrations. The data are presented as box plots, showing the distribution of the lipid accumulation at each ZnO concentration. The letters on top of each box plot show the statistical differences.

As illustrated in Figure 6, the lipid content of *C. vulgaris* under varying concentrations of ZnO NPs reveals a concentration-dependent response, emphasizing its potential for biofuel production. In the control group, lipid accumulation was minimal (13.8%), reflecting the standard metabolic activity in the absence of stress. ZnO NP exposure between 20 and 50 mg/L significantly enhanced lipid content, with a peak value of 48% at 50 mg/L. This suggests that ZnO NPs induce oxidative stress, redirecting metabolic activity toward lipid biosynthesis, a well-documented adaptive response in microalgae under stress conditions.^32−34^

At 20 mg/L ZnO, the lipid content rose to 33.7%, indicating that mild oxidative stress effectively stimulates lipid accumulation. The trend continued with further increases at 30 mg/L (40.5%) and 40 mg/L (45.3%), reflecting the optimal balance between stress induction and cellular functionality. These moderate ZnO NP concentrations appear to activate lipid biosynthesis without significantly impairing growth or photosynthetic activity, aligning with the findings of enhanced enzymatic responses and pigment retention.

However, at 100 mg/L ZnO NPs, the lipid content declined sharply to 30%, despite elevated oxidative stress levels indicated by previous catalase activity results. This reduction points to a disruption in lipid biosynthesis pathways due to excessive stress, which likely impacts photosynthesis, pigment stability, and overall cellular health. Moreover, high NP concentrations can induce lipid peroxidation, impairing cellular function and causing alterations in cell membranes, thus leading to a loss of membrane selectivity and integrity.^31^ The observed decline underscores the critical threshold beyond which oxidative stress transitions from being beneficial (stimulating lipid production) to being detrimental (compromising cell viability).

These findings establish that ZnO NPs are effective at promoting lipid accumulation in *C. vulgaris* at moderate concentrations (20–50 mg/L), making this range highly suitable for biofuel applications. Within this range, enhanced lipid biosynthesis occurs without significant detriments to biomass or pigment quality. However, concentrations beyond 50 mg/L lead to excessive oxidative stress, reducing lipid yields and limiting the utility of the biomass for biofuel production. Future studies should focus on the molecular mechanisms underlying lipid induction and explore interventions, such as antioxidant supplementation, to support lipid productivity at higher ZnO NP concentrations.

Figure 6 illustrates the lipid content of *C. vulgaris* under varying concentrations of ZnO nanoparticles. As can be seen, the lipid content of *C. vulgaris* increases in a concentration-dependent manner when exposed to varying levels of ZnO nanoparticles. In the control group, the lipid content is 13.8%. At 20 mg/L ZnO, the lipid content rises to 33.7%, indicating that a moderate level of ZnO nanoparticles can effectively stimulate lipid accumulation in the microalgae.

The lipid content continues to increase at higher ZnO concentrations, reaching a peak of 45.3% at 40 mg/L. This suggests that the optimal balance between stress induction and cellular functionality occurs in the 20–50 mg/L range of ZnO nanoparticles. However, at the highest concentration of 100 mg/L ZnO, the lipid content drops sharply to 30%. One explanation for this could be that excessive oxidative stress from high nanoparticle levels can disrupt lipid biosynthesis pathways and compromise cellular function. Thus, the reported data support the conclusion that moderate ZnO nanoparticle concentrations (20–50 mg/L) are highly suitable for biofuel applications, as they effectively stimulate lipid production without significantly impairing growth or photosynthetic activity.

### XPS Analysis of the Interaction

**Figure 7:**
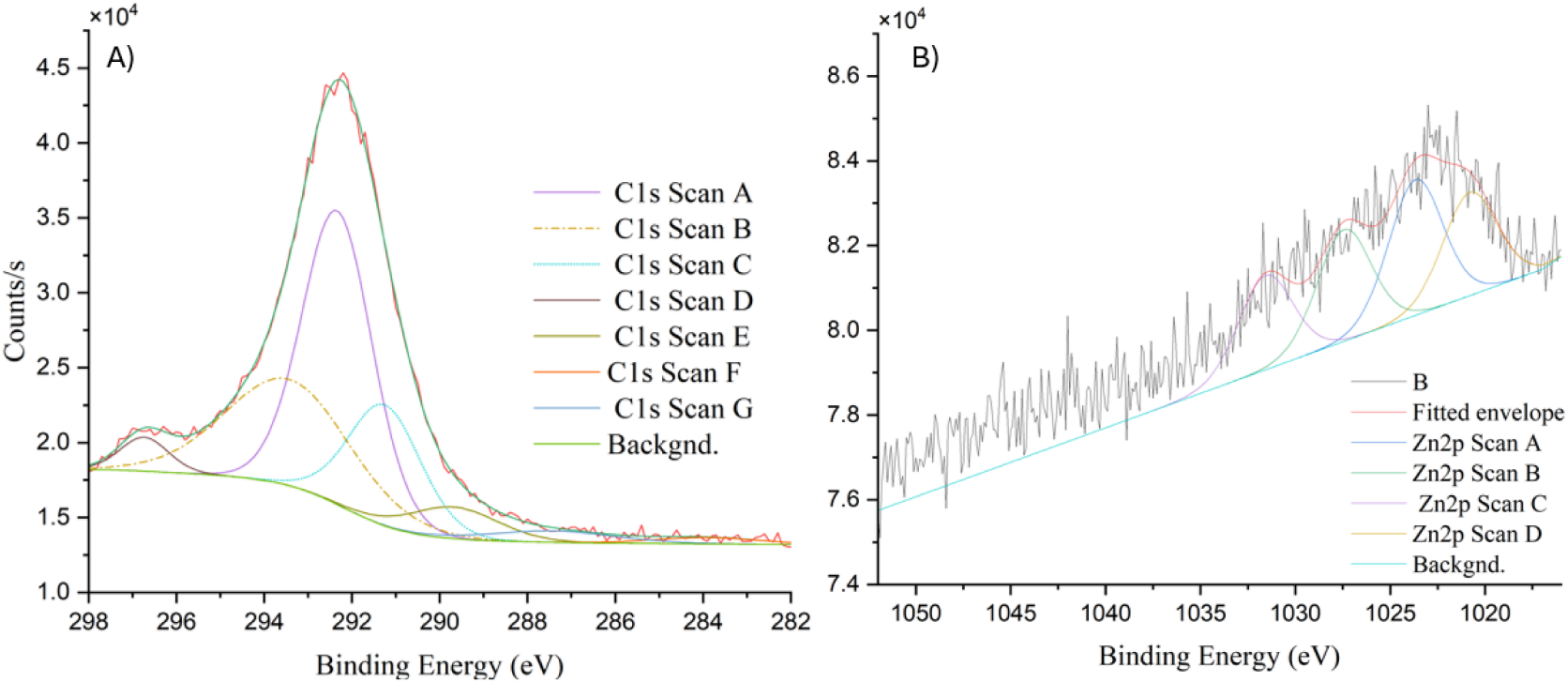
High-resolution XPS spectrum of (A) the C 1s region, showing peaks corresponding to C–C/C–H (∼284.8 eV), C–O (∼286.5 eV), and C=O (∼288.5 eV) and (B) the Zn 2p region, displaying Zn 2p3/2 (∼1021.5 eV) and Zn 2p1/2 (∼1044.5 eV) peaks.

The XPS scan for carbon (Figure 7A) revealed multiple peaks corresponding to distinct binding energies associated with various functional groups in one representative treatment of 50 mg/L ZnO NPs. These peaks include contributions from C–C and C–H bonds (∼284.8 eV), C–O bonds (∼286.5 eV), and C=O bonds (∼288.5 eV). The observed C–O and C=O signals are indicative of the presence of oxygen-containing functional groups, such as hydroxyl and carboxyl, on the surface of the *C. vulgaris* cells. These groups are essential for interacting with ZnO NPs, as they can facilitate adsorption and binding through electrostatic interactions or coordination bonds.

The increased intensity of these oxygenated carbon peaks suggests a potential modification or interaction between ZnO NPs and the algal cell surface, as the nanoparticles may induce oxidative stress, leading to the generation of ROS and subsequent modification of the cell wall. Additionally, the distribution of these carbon species reflects the biochemical composition of the algal cell wall, which plays a crucial role in ZnO NP binding.

The XPS scan for zinc (Figure 7B) shows prominent peaks associated with Zn 2p1/2 and Zn 2p3/2 at binding energies around ∼1021.5 and ∼1044.5 eV, respectively. These peaks confirm the presence of ZnO nanoparticles interacting with algal cells. The deconvolution of the Zn peaks reveals the presence of ZnO and possible Zn-organic complexes, indicating that ZnO NPs have adhered to or reacted with the algal surface. This phenomenon was also observed in the microalga *Coelastrella terrestris*, in which their cell wall induces the aggregation of ZnO NPs around their cells, reducing light availability to the algal cells.^35^ In *Chlorella* sp. cells, it has been proposed that cellulose, polysaccharides, and glycoproteins induce a negative charge in the cell wall, which can interact with the positive charge in Zn ions, thus facilitating their interaction.^36^

The interaction between ZnO NPs and *C. vulgaris* is further evidenced by the broadening of the Zn 2p peaks, which may result from surface modification or coordination with the functional groups present on the algal cell wall. The presence of Zn-organic complexes aligns with the hypothesis that the carboxyl and hydroxyl groups identified in the carbon XPS scan play significant roles in binding ZnO NPs.

Interaction Mechanism: The results indicate that ZnO nanoparticles interact with the algal cell wall primarily through surface functional groups, such as hydroxyl (−OH) and carboxyl (−COOH). These groups not only facilitate adsorption but also provide sites for complex formation with zinc ions released from ZnO NPs under environmental conditions. The interaction may lead to the partial dissolution of ZnO, releasing Zn ions that can further enhance toxicity through ionic stress and oxidative damage. However, the strong binding of ZnO to the cell wall may also limit internalization, suggesting a surface-dominated interaction.

Biological Implications: The XPS data support the hypothesis that ZnO NPs can induce biochemical and structural changes in *C. vulgaris*. While the algal cell wall provides a barrier to nanoparticle internalization, the observed surface interactions may still trigger stress responses, including oxidative stress, which can alter cellular metabolism and growth. The ability of ZnO NPs to bind to functional groups on the algal surface highlights their potential for bioremediation applications, where interactions with cell walls can be leveraged for nanoparticle immobilization or pollutant capture.

In summary, the XPS analysis demonstrates that ZnO nanoparticles interact with *C. vulgaris* through oxygen-containing functional groups on the cell wall, leading to the formation of Zn-organic complexes and potential surface modification. This interaction has significant implications for understanding the environmental behavior and toxicity of ZnO NPs in aquatic ecosystems.

### Biofuel Suitability Score Analysis

**Figure 8:**
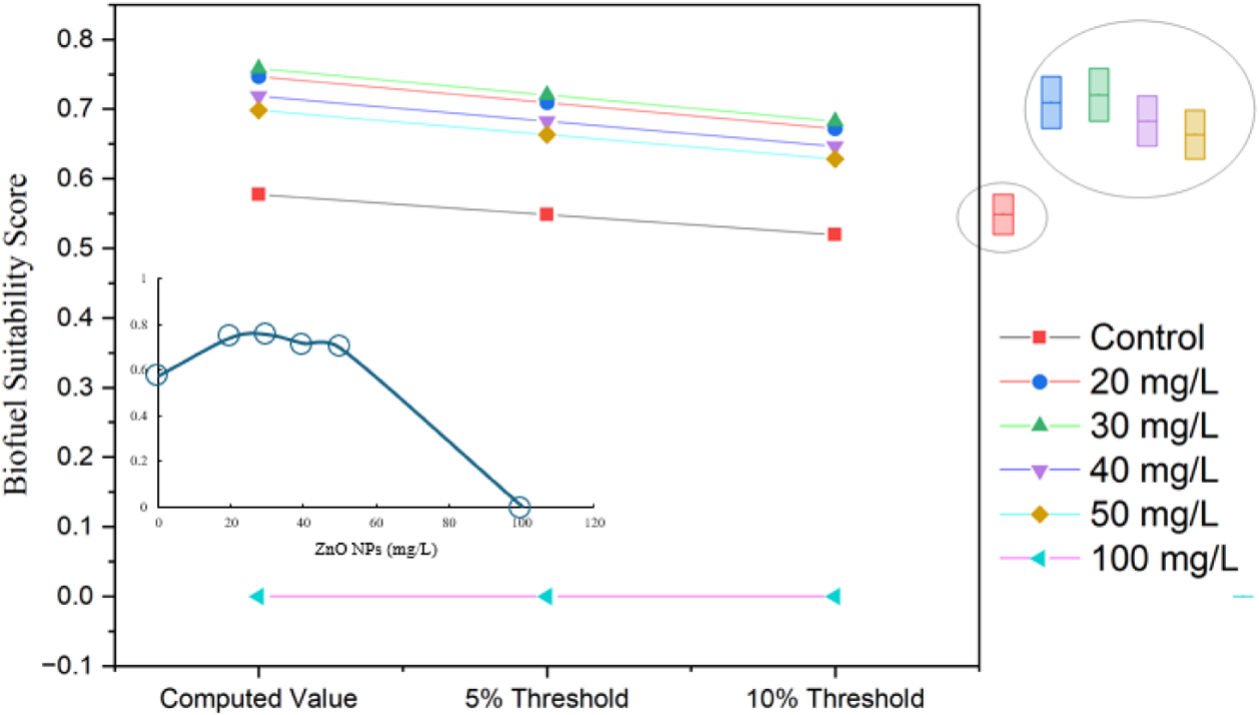
Marginal box plot illustrating the biofuel suitability score of *C. vulgaris* exposed to different concentrations of ZnO NPs. The computed values (left) represent the biofuel suitability score at each treatment level, while the 5% and 10% thresholds (bottom) indicate the acceptable deviation range for biofuel optimization. The inset graph provides a detailed trend of the biofuel suitability score as a function of the ZnO NP concentration. The marginal distributions (right) visualize the data spread across different ZnO NP treatments.

Figure 8 depicts the Biofuel Suitability Score (BSS) of the microalgae *C. vulgaris* under varying concentrations of ZnO NPs. The BSS is a metric that reflects the combined effects of lipid accumulation, biomass production, pigment content, and oxidative stress in the algae.

As can be seen, the BSS curve reaches a peak at moderate ZnO NP concentrations, specifically in the range of 30–50 mg/L. This indicates an optimal range for biofuel production from *C. vulgaris*, as it represents a balance between enhanced lipid accumulation, relatively stable biomass production, and manageable levels of oxidative stress. At lower ZnO NP concentrations (0–20 mg/L), the BSS increases steadily. This is because the mild oxidative stress induced by the low nanoparticle levels helps to enhance lipid accumulation, while maintaining the biomass and pigment content of the algae. Beyond 50 mg/L ZnO NPs, the BSS begins to decline sharply. This is due to the intensification of oxidative stress, as evidenced by elevated catalase activity and decreased pigment and carotenoid levels in the algae. At the highest tested concentration of 100 mg/L ZnO NPs, the BSS drops to zero. This indicates that severe oxidative stress overwhelms the cells, leading to a reduction in lipid content and impairment of photosynthetic efficiency. As a result, this concentration is considered unsuitable for biofuel production from *C. vulgaris*.

The inset plot in the figure provides additional insights into the variability and threshold levels of the BSS under different ZnO NP concentrations. The marginal box charts highlight the importance of carefully modulating the nanoparticle levels to optimize biofuel suitability, as higher concentrations contribute to increased fluctuations and potential instability in the biofuel production process.

## Conclusion

This study provides a comprehensive evaluation of the multifaceted impacts of ZnO NPs on *C. vulgaris*, focusing on lipid accumulation, biomass productivity, and stress responses to optimize biofuel production. Key findings indicate that ZnO NPs induce oxidative stress in a concentration-dependent manner, which can be leveraged to enhance lipid biosynthesis, a critical determinant of biodiesel feedstock. Moderate ZnO NP concentrations (20–50 mg/L) were identified as the optimal range for balancing stress-induced lipid production with cellular health, achieving peak lipid content (48%) and maximizing the Biofuel Suitability Score (BSS).

Higher ZnO NP concentrations (above 50 mg/L) resulted in excessive oxidative stress, as evidenced by significant increases in catalase activity, reductions in pigment content, and declines in the lipid yield. These findings underscore the threshold beyond which ZnO-induced stress becomes detrimental, highlighting the need for precise control of nanoparticle exposure to maintain biomass quality and biofuel potential. This work advances our understanding of nanoparticle–algal interactions and establishes a scalable framework for integrating nanotechnology into sustainable energy production. Future research should explore strategies such as antioxidant supplementation and alternative nanoparticle formulations to enhance microalgal resilience and sustain high lipid productivity under varying environmental conditions. This study serves as a foundation for optimizing biofuel systems through targeted stress management in microalgae.
